# Maternal diabetes as a prenatal risk factor for multiple sclerosis in offspring: a systematic review and meta-analysis

**DOI:** 10.1007/s10072-026-09367-9

**Published:** 2026-09-02

**Authors:** Mehdi Mohammadifar, Mahyar Ghajarzdaeh, Mahsa Ghajarzadeh

**Affiliations:** 1 Mallinckrodt Institute of Radiology, Department of Radiology , Washington University School of Medicine in St. Louis, St. Louis, MO USA; 2 Shahid Beheshti University, Tehran, Iran

**Keywords:** Multiple sclerosis, Diabetes, Gestational, Maternal exposure, Risk

## Abstract

**Background:**

Multiple sclerosis (MS) is a chronic demyelinating disease of the central nervous system with a complex etiology involving genetic and environmental factors. Maternal diabetes during pregnancy has been hypothesized to influence offspring MS risk through intrauterine metabolic programming, yet the evidence remains inconclusive.

**Objective:**

To systematically review and meta-analyze the association between maternal diabetes and the risk of MS in offspring.

**Methods:**

A systematic literature search was conducted across PubMed, Scopus, Web of Science, and Embase, identifying 427 records. After removing 188 duplicates, 239 records were screened, 38 full-text reports were assessed for eligibility, and 4 studies met the inclusion criteria. Pooled risk ratios (RR) were calculated using both common-effect and random-effects models. Heterogeneity was assessed using the I² statistic, and publication bias was evaluated using Egger’s and Begg’s tests.

**Results:**

The studies, published between 2009 and 2026, were conducted in Denmark, the USA (two studies), and Norway, utilizing diverse designs including nationwide register-based cohorts and a case-control study. The common-effect model yielded a pooled RR of 1.42 (95% CI: 1.09–1.85), while the random-effects model produced a non-significant pooled RR of 3.13 (95% CI: 0.68–14.30). Substantial heterogeneity was observed across studies (I² = 95.2%, τ² = 2.2357, *p* < 0.0001). Individual study risk ratios ranged from 0.91 (95% CI: 0.60–1.38) to 28.35 (95% CI: 13.33–60.29). Neither Egger’s test (*p* = 0.4364) nor Begg’s test (*p* = 0.1742) indicated significant publication bias, though these tests have limited power with fewer than ten studies.

**Conclusion:**

This meta-analysis did not demonstrate a statistically significant association between maternal diabetes and MS risk in offspring, likely owing to substantial between-study heterogeneity and the limited number of included studies. Large, well-characterized prospective cohorts are needed to clarify this relationship, elucidate underlying mechanisms, and inform preventive strategies.

## Introduction

One of the most common leading causes of disability worldwide is multiple sclerosis (MS) [1], with complex pathophysiology characterized by inflammatory processes, immune dysregulation, oxidative stress, microglial activation, and altered vascular permeability [2] which arises from chronic immune dysregulation driven by complex interactions between genetic and environmental factors [3]. Genetics play a predominant role in development of MS(concordance in monozygotic twins is estimated at 25%–30%), and 5%-14% for dizygotic twins [4, 5]. Alternatively, influence and timing of different environmental factors such as smoking exposure, Epstein-Barr virus infection, and habitat latitude in MS development are not very clear [6]. It is also unclear how early-life factors around the time of gestation may contribute to MS development. For instance, maternal vitamin D levels and being born large for gestational age (LGA) have been associated with an increased risk of developing MS in offspring [7, 8]. Adverse pregnancy outcomes, through immune system dysregulation, may increase the risk of a wide range of diseases in offspring, including cardiovascular and metabolic conditions, as well as neurodegenerative disorders such as multiple sclerosis [8–10]. Both pregestational and gestational diabetes in mothers are associated with increased risks of congenital, metabolic, cardiovascular, and neurodevelopmental conditions in offspring, with the degree of risk depending on when and how severely hyperglycemia occurs [11, 12].

The mechanisms linking maternal diabetes to offspring MS risk are not fully understood, but two pathways have been proposed. First, maternal hyperglycemia may alter fetal immune development through epigenetic changes, predisposing to later autoimmune dysfunction. Second, it may indirectly increase risk by promoting higher birth weight and childhood obesity, both associated with MS through inflammation and reduced vitamin D levels. Notably, pregestational diabetes shows stronger effects, highlighting early pregnancy as a critical window for disease programming [7].

To date, several studies have investigated the association between maternal diabetes and MS risk, so this systematic review and meta-analysis aims to summarize their findings.

## Methods

We followed the Preferred Reporting Items for Systematic Reviews and Meta-Analyses (PRISMA) 2020 guidelines in conducting and reporting this systematic review and meta-analysis [13].

### Inclusion criteria

Cross-sectional, case–control, and cohort studies. Studies which are published in any languages.

### Exclusion criteria

Studies lacking sufficient data, as well as case reports, case series, and review articles, were excluded.

## Search strategy

wo independent researchers conducted a comprehensive search of PubMed, Scopus, EMBASE, PsycINFO, Web of Science, and Google Scholar on February 1, 2026. Gray literature, including reference lists of included studies, theses, and conference abstracts, was also reviewed.

### PubMed search strategy was

(((((((((((“Diabetes Mellitus“[MeSH Terms]) OR (“Gestational Diabetes“[MeSH Terms])) OR (Maternal Diabetes[Text Word])) OR (Diabetes in Pregnancy[Text Word])) OR (Pregestational Diabetes[Text Word])) OR (Pre-gestational Diabetes[Text Word])) OR (Gestational Diabetes[Text Word])) OR (Hyperglycemia in Pregnancy[Text Word])) OR (Maternal Hyperglycemia[Text Word])) OR (Diabetic Pregnancy[Text Word])) OR (Diabetes, Maternal[Text Word])) AND ((((((Multiple Sclerosis[MeSH Terms]) OR (Multiple Sclerosis[Text Word])) OR (Sclerosis, Multiple[Text Word])) OR (Sclerosis, Disseminated[Text Word])) OR (Disseminated Sclerosis[Text Word])) OR (Multiple Sclerosis, Acute Fulminating[Text Word])))

((((((((((((Prenatal Exposure Delayed Effects[MeSH Terms]) OR (Prenatal Exposure[Text Word])) OR (Prenatal Exposur*[Text Word])) OR (In Utero Exposure[Text Word])) OR (Fetal Exposure[Text Word])) OR (Maternal Exposure[Text Word])) OR (Perinatal Exposure[Text Word])) OR (Early Life Exposure[Text Word])) OR (Gestational Exposure[Text Word])) OR (Intrauterine Exposure[Text Word])) OR (Antenatal Exposure[Text Word])))

AND

((((((Multiple Sclerosis[MeSH Terms]) OR (Multiple Sclerosis[Text Word])) OR (Sclerosis, Multiple[Text Word])) OR (Sclerosis, Disseminated[Text Word])) OR (Disseminated Sclerosis[Text Word])) OR (Multiple Sclerosis, Acute Fulminating[Text Word])))

## Selection process and data collection

All retrieved records were imported into EndNote, and duplicates were removed. Titles and abstracts were screened, followed by full-text assessment of eligible studies. Relevant data were extracted from each study and recorded in an Excel file. Any disagreements between the two reviewers were resolved by a third reviewer.

## Data items

Two independent reviewers extracted data from each included study, including the first author’s name, publication year, country of origin, disease duration, type of maternal diabetes, study design, mean age of participants, sex distribution, number of pregnancies with and without diabetes, and the number of offspring who developed multiple sclerosis in both the diabetic and non-diabetic groups.

## Study risk of bias assessment

The risk of bias of the included studies was assessed independently by two reviewers using the Newcastle–Ottawa Scale (NOS) for cohort and case–control studies [14].The NOS is a widely used tool for evaluating the methodological quality of non-randomized studies in systematic reviews. For cohort and case–control studies, the scale assesses three domains: selection of study groups, comparability of groups, and ascertainment of exposure or outcome. For cross-sectional studies, the assessment includes selection of participants, comparability between groups, and outcome evaluation. Studies were assigned a quality score based on the NOS criteria, with higher scores indicating better methodological quality. Studies scoring more than 6 points were considered high quality, those scoring 2–6 points were considered of fair quality, and those scoring 1 point or less were considered poor quality.

## Data synthesis

All statistical analyses were done using R (Version 4.4.1). Meta, metafor, and forest plot packages were used [15].

Statistical heterogeneity among studies was assessed using the I² statistic. An I² value greater than 50% was considered indicative of substantial heterogeneity. Therefore, pooled relative risks (RRs) and their corresponding 95% confidence intervals (CIs) were estimated using the inverse-variance method under a random-effects model. We also evaluated publication bias using funnel plot, Egger and Begg’s tests.

## Certainty assessment

For all estimated effect sizes, we reported 95%CI.

## Results

**Fig. 1:**
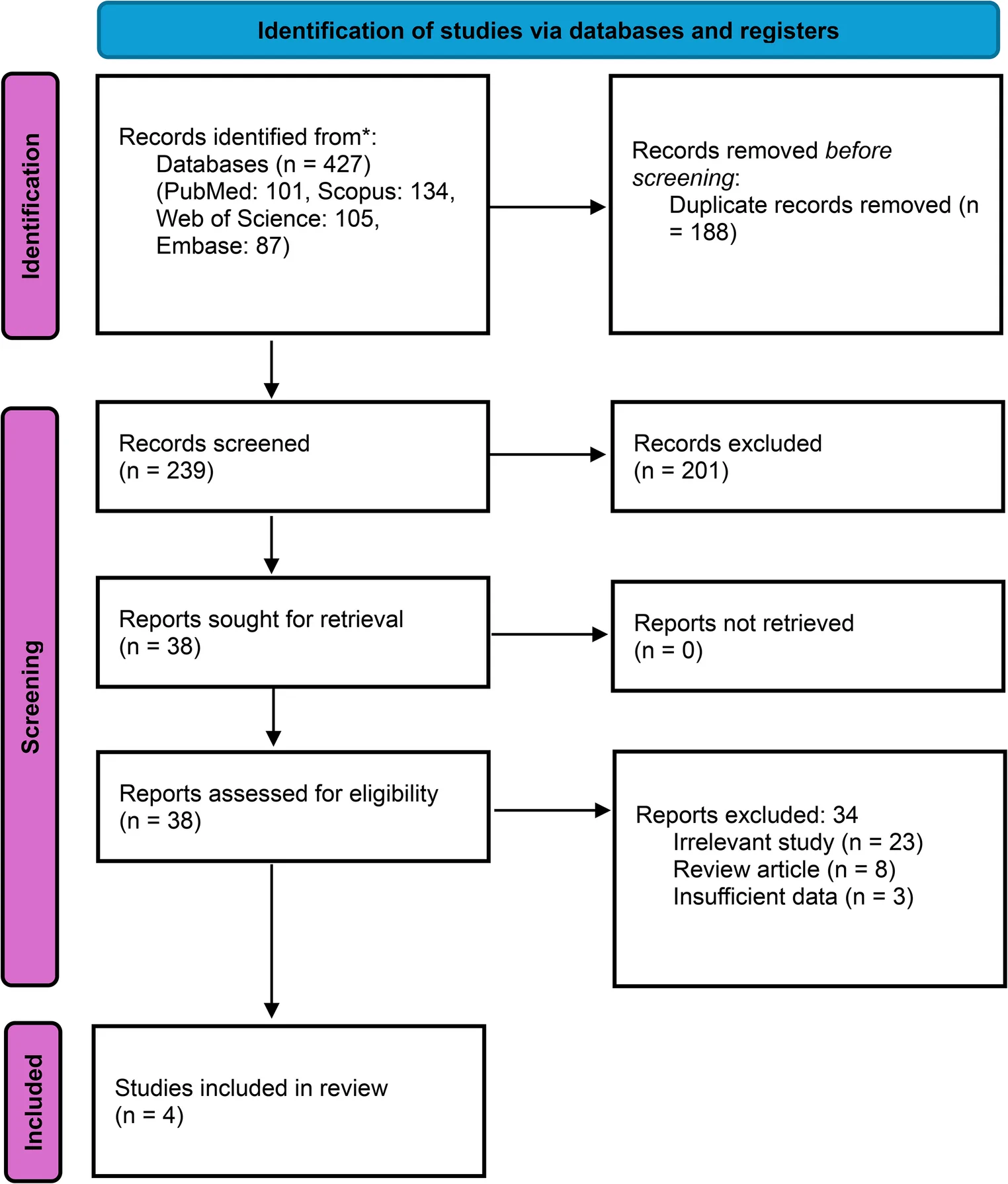
Flow diagram summarizing the selection of eligible studies

The literature search revealed 427 articles, after deleting duplicates 86 articles remained. Four studies were included for meta-analysis (Fig. 1).

The studies, published between 2009 and 2026, were conducted in Denmark, the USA (two studies), and Norway, utilizing diverse designs including nationwide register-based cohorts and a case-control study. They encompassed a wide range of offspring ages from pediatric to adult populations and varied in exposure assessment (pregestational, gestational, or any maternal diabetes (Table 1).

**Table 1:** Data extracted from included studies

First author	Publication year	Country	Duration of disease	Type of diabetes	Design	Mean age	Gender	Diabetic /non-diabetic cases	MS in diabetic/non-diabetic
Nielsen et al (16).	2021 (received 2020)	Denmark	Follow-up to 2018 (births 1978–2008)	Pregestational DM (mainly); Gestational DM	Nationwide register-based cohort	Not specified (offspring followed from age 10)	Mixed (MS: 1634 F, 698 M total)	Diabetes = 17,001 No diabetes = 1,618767	MS in maternal DM = 22, MS in non diabetic mothers = 2310
Gardener et al (17).	2009	USA	Not specified (adult nurses; cases pre/post baseline)	Diabetes during pregnancy (maternal report)	Prospective cohort (Nurses’ Health Studies + mothers’ subcohort)	Adult nurses (30–55 at baseline)	Female only (nurses)	Diabetes = 160 Nondiabetes = 34,012	MS in maternal DM = 2 MS in non diabeteic mothers = 173
Graves et al (18).	2017	USA	Pediatric-onset (cases within 4 years of onset)	Maternal diabetes (incl. gestational)	Case-control (pediatric MS network)	Cases: median 15.7 yrs; Controls: 14.6 yrs	Mixed (62% F cases, 54% F controls)	DM = 20 NONDM = 567	MS in maternal diabetic = 10, MS in non diabetic mothers = 10
Wolfova et al [7].	2026	Norway	Adult-onset (follow-up 2009–2019; births 1967–1989)	Maternal diabetes (type 2, gestational, etc.)	Nationwide closed cohort (register-based)	Adult (≥ 18 at follow-up start)	Mixed (51.2% male in cohort)	Diabetes = 2662 Non = 1,165,552	MS in maternal diabetes = 21, MS in non = 5757

All included studies had high quality which is summarized in Table 2.

**Table 2:** Quality assessment of included studies

Study	Design	S1	S2	S3	S4	C1	C2	O/E1	O/E2	O/E3	Total
Nielsen et al. (2021)	Cohort	*	*	*	*	*	0	*	*	0	7
Gardener et al. (2009)	Cohort	*	*	0	*	*	0	*	*	0	6
Graves et al. (2017)	Case-control	*	*	*	*	*	0	*	*	0	7
Wolfova et al. (2026)	Cohort	*	*	*	0	*	*	*	*	*	8

NOS criteria for cohort studies: S1, representativeness of the exposed cohort; S2, selection of the non-exposed cohort; S3, ascertainment of exposure; S4, demonstration that the outcome was not present at the start; C1-C2, comparability based on design or analysis; O1, assessment of outcome; O2, follow-up long enough for the outcome to occur; O3, adequacy of follow-up

**Fig. 2:**
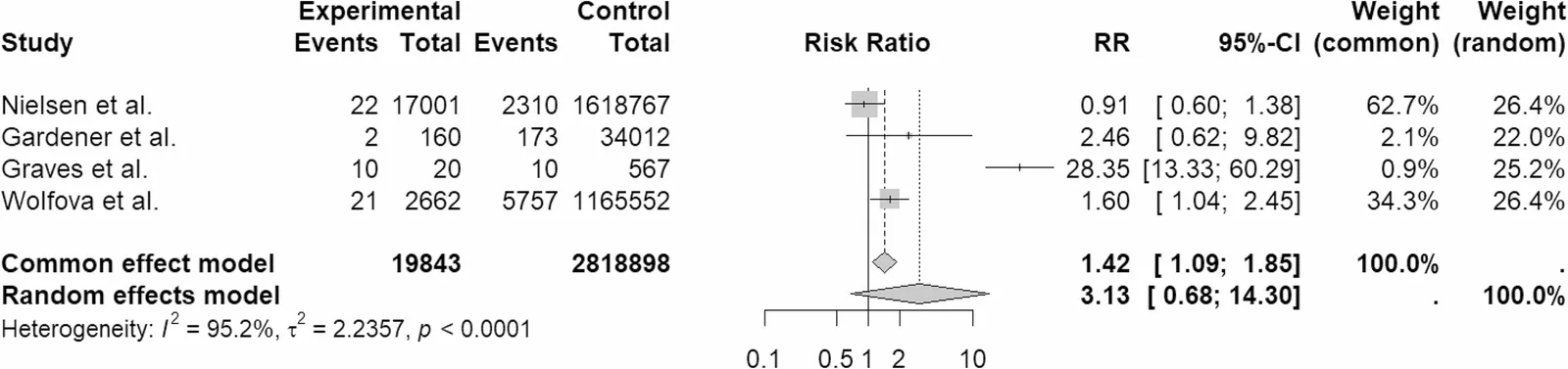
Forest plot showing the pooled estimate

A total of four studies comprising 2,818,361 participants were included in the meta-analysis evaluating the association between maternal diabetes and the risk of multiple sclerosis (MS) in offspring.In the random-effects meta-analysis, the association between maternal diabetes and offspring risk of multiple sclerosis (MS) varied considerably across studies. Nielsen et al. reported no significant association (RR, 0.91; 95% CI, 0.60–1.38), whereas Gardener et al. observed an elevated but non-significant risk (RR, 2.46; 95% CI, 0.62–9.82). Graves et al. reported the strongest association, with offspring of diabetic mothers demonstrating a markedly increased risk of MS (RR, 28.35; 95% CI, 13.33–60.29), although this estimate was based on a small sample size. Wolfova et al. found a significant 60% increase in MS risk among exposed offspring (RR, 1.60; 95% CI, 1.04–2.45). When these studies were pooled using a random-effects model, maternal diabetes was associated with a non-significant increase in offspring MS risk (pooled RR, 3.13; 95% CI, 0.68–14.30). The substantial between-study heterogeneity (I² = 95.2%, *p* < 0.0001) indicates considerable variability in effect estimates across studies (Fig. 2).

Publication bias and small-study effects were assessed using a funnel plot, Egger’s test, and Begg’s test. Given the limited number of included studies, these analyses were considered exploratory because their statistical power is reduced when fewer than 10 studies are available. They were performed to examine potential funnel plot asymmetry and to provide a transparent evaluation of possible publication bias. Accordingly, the findings were interpreted with caution and were not considered sufficient to rule out publication bias.

**Fig. 3:**
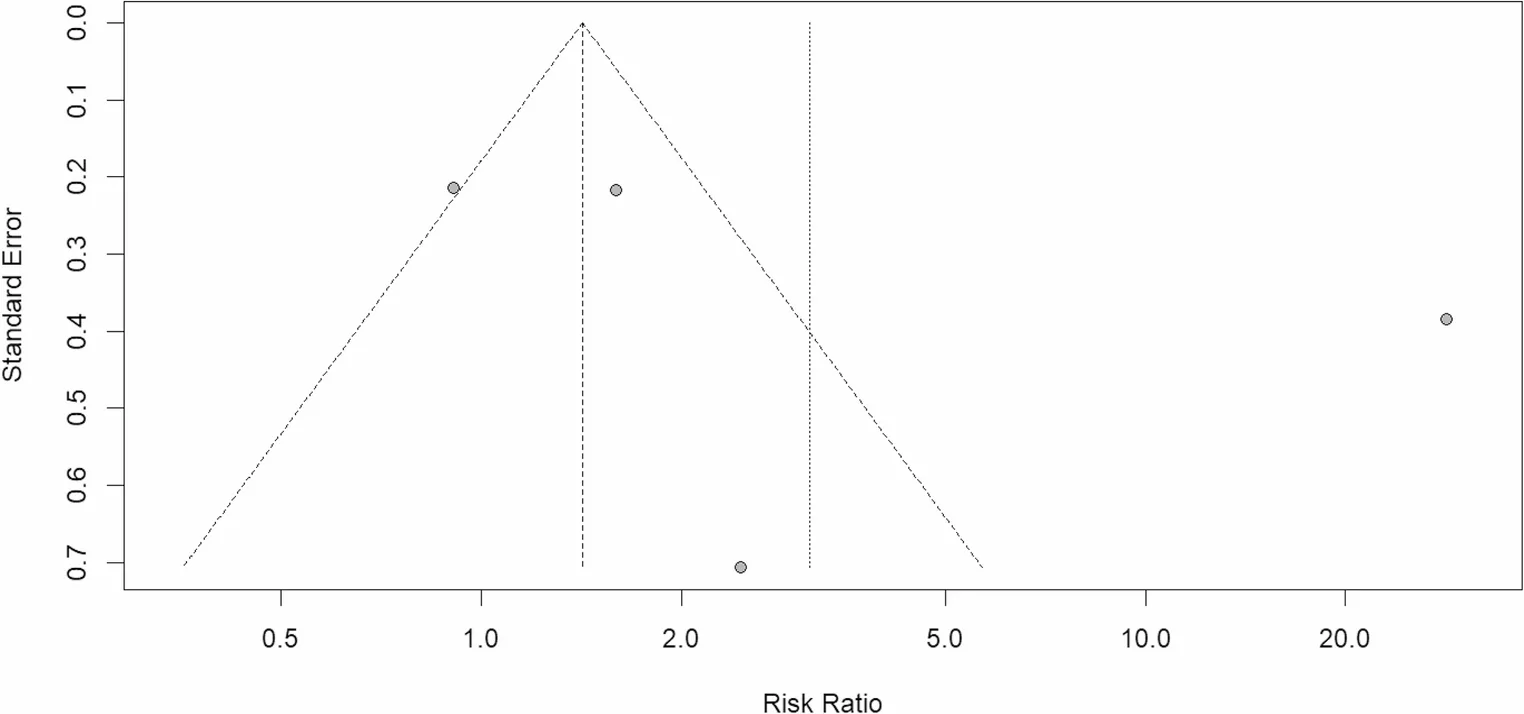
Funnel plot showing no publication bias

Funnel plot showing no evidence of publication bias (Fig. 3).

Egger’s test (t = 0.96, df = 2, p-value = 0.4364), Begg’s test (z = 1.36, SE = 2.9, p-value = 0.1742).

## Discussion

This systematic review and meta-analysis synthesized evidence from four observational studies involving more than 2.8 million participants to investigate the association between maternal diabetes and the risk of MS in offspring. The pooled analysis using a common-effect model demonstrated a statistically significant 42% increased risk of MS among offspring of mothers with diabetes (RR, 1.42; 95% CI, 1.09–1.85). In contrast, the random-effects model yielded a non-significant pooled risk ratio of 3.13 (95% CI, 0.68–14.30). The discrepancy between the two models is likely attributable to the substantial between-study heterogeneity (I² = 95.2%, *p* < 0.001), indicating considerable variability in study populations, diabetes definitions, and methodological approaches. Therefore, the findings should be interpreted with caution, although the overall evidence suggests a potential association between maternal diabetes during pregnancy and an increased risk of MS in offspring.

The association between maternal diabetes and offspring risk of MS was generally positive across the included studies, although the magnitude of the effect varied considerably. Nielsen et al. [16], in a large nationwide Danish cohort, reported a non-significant increase in risk among offspring of mothers with pregestational diabetes (HR, 2.25; 95% CI, 1.35–3.75), while no association was observed for gestational diabetes. In the United States, Gardener et al. [17] reported an increased risk of MS among offspring exposed to maternal diabetes during pregnancy (RR, 10.2; 95% CI, 2.5–42.0), although this estimate was based on a very small number of exposed cases. Similarly, Graves et al. [18] found that maternal illness during pregnancy was associated with an increased risk of pediatric-onset MS, although maternal diabetes itself was not identified as a significant independent risk factor in the primary analysis. More recently, Wolfova et al. [7] demonstrated that maternal diabetes was associated with a more than twofold increased risk of adult-onset MS in offspring (HR, 2.15; 95% CI, 1.37–3.37) in a nationwide Norwegian cohort. Taken together, these findings suggest a potential association between maternal diabetes and MS susceptibility in offspring; however, the pooled RR showed no significant association between maternal diabetes and offspring MS development.

The findings of the present meta-analysis are generally consistent with previous evidence suggesting a link between diabetes and the development of MS. In a nationwide Danish cohort study, Nielsen et al. [16] reported that offspring of mothers with pre-gestational diabetes had a significantly increased risk of developing MS (HR, 2.25; 95% CI, 1.35–3.75), whereas no significant association was observed for gestational diabetes (HR, 1.03; 95% CI, 0.49–2.16). Importantly, paternal diabetes was not associated with MS risk in offspring, supporting the hypothesis that the intrauterine metabolic environment, rather than shared genetic susceptibility alone, may contribute to disease development [19]. Furthermore, a recent meta-analysis by Tang et al. [20] demonstrated that diabetes mellitus was associated with an increased risk of MS overall (HR, 1.59; 95% CI, 1.24–2.05), with type 2 diabetes showing a particularly significant association (HR, 1.46; 95% CI, 1.10–1.94). Collectively, these findings support a potential role of maternal metabolic dysregulation during pregnancy in shaping long-term neuroimmune susceptibility in offspring.

Several biological mechanisms may explain the observed association between maternal diabetes and the risk of MS in offspring [7]. Maternal hyperglycemia during pregnancy can alter the intrauterine environment and influence fetal immune system development during critical periods of immune programming. Such prenatal metabolic disturbances may induce long-lasting immunological changes that predispose offspring to chronic immune dysregulation, a hallmark of MS pathogenesis. Furthermore, maternal diabetes has been associated with increased birth weight and a higher risk of childhood obesity, both of which may contribute to subsequent MS susceptibility. Childhood and adolescent obesity are well-established risk factors for MS and have been associated with approximately a twofold increase in disease risk [7, 21]. Mendelian randomization studies further support a causal role of childhood obesity and vitamin D deficiency in MS development. Proposed mechanisms linking obesity to MS include chronic low-grade systemic inflammation, dysregulated adipokine secretion, insulin resistance, and reduced circulating vitamin D levels [22]. However, mediation analyses suggest that only a small proportion of the association between obesity and MS is explained by vitamin D deficiency, indicating that additional metabolic and immunological pathways are likely involved [22]. Collectively, these findings support the hypothesis that adverse metabolic exposures during fetal life may contribute to long-term neuroimmune vulnerability and increase the risk of MS in later life.

The considerable heterogeneity observed in this meta-analysis (I² = 95.2%) should be taken into account when interpreting the pooled estimates. Several factors may have contributed to this variability. First, the definition of maternal diabetes differed across studies, with some investigations evaluating pregestational diabetes, others gestational diabetes, and others combining all forms of maternal diabetes into a single exposure category. This distinction is clinically important, as the timing, severity, and duration of fetal exposure to maternal hyperglycemia may differ substantially between diabetes subtypes. Second, the included studies employed diverse methodological approaches, ranging from nationwide population-based cohort studies using registry data to case–control and prospective cohort designs relying on maternal self-report, which may have introduced differences in exposure ascertainment and outcome assessment. Third, the populations studied varied considerably with respect to age at MS onset, geographic location, and healthcare context. The studies were conducted in Denmark, Norway, and the United States, countries that differ in the prevalence of diabetes and MS, genetic background, environmental exposures, and healthcare practices. Although exploration of heterogeneity through subgroup analyses or meta-regression is generally recommended when substantial heterogeneity is present, such analyses were not feasible in the current study because only four studies met the inclusion criteria. Furthermore, statistical tests for heterogeneity have limited power when the number of included studies is small. Therefore, the pooled estimates should be interpreted cautiously, and additional well-designed studies are needed to clarify the nature and magnitude of the association between maternal diabetes and offspring MS risk.

Publication bias was assessed using both Egger’s test (*p* = 0.4364) and Begg’s test (*p* = 0.1742), neither of which indicated significant asymmetry. However, these tests have limited statistical power when fewer than ten studies are included, and the absence of significant publication bias should be interpreted cautiously.

Several limitations of this meta-analysis should be considered when interpreting the findings. First, only four studies met the inclusion criteria, limiting the statistical power of the analysis and restricting the ability to investigate potential sources of heterogeneity through subgroup analyses or meta-regression. Second, the number of MS cases among offspring exposed to maternal diabetes was relatively small across the included studies, resulting in wide confidence intervals and reduced precision of individual effect estimates. Third, substantial between-study heterogeneity was observed, likely reflecting differences in study design, exposure definitions, outcome ascertainment, and population characteristics. In addition, residual confounding cannot be excluded. Important factors associated with MS risk, including maternal obesity, smoking during pregnancy, vitamin D status, Epstein–Barr virus exposure, genetic susceptibility, and other environmental influences, were not consistently measured or adjusted for across studies. Furthermore, the observational nature of all included studies precludes causal inference and leaves the possibility of unmeasured confounding and selection bias. Finally, publication bias could not be formally assessed because fewer than 10 studies were available for analysis, making conventional methods such as funnel plots and Egger’s or Begg’s tests unreliable. Therefore, the findings should be interpreted cautiously until confirmed by larger, well-designed prospective studies.

Despite these limitations, the present meta-analysis contributes to the growing body of evidence suggesting that the prenatal metabolic environment may play a role in shaping long-term susceptibility to multiple sclerosis (MS). These observations support the hypothesis that maternal metabolic disturbances during pregnancy may influence fetal immune development and subsequent neuroimmune vulnerability. Future studies should seek to determine whether the observed association is primarily attributable to pregestational diabetes, gestational diabetes, or both, and to clarify the potential mediating roles of birth weight, childhood obesity, and other early-life metabolic factors. In addition, mechanistic research is needed to elucidate the biological pathways through which maternal hyperglycemia may alter immune system programming and increase MS susceptibility. Large prospective cohort studies with detailed information on diabetes subtype, glycemic control, treatment, and relevant confounding factors will be essential to strengthen causal inference and inform potential preventive strategies.

## Conclusions

In this systematic review and meta-analysis, maternal diabetes was associated with an increased risk of multiple sclerosis (MS) in offspring in the common-effect model; however, the association did not remain statistically significant in the random-effects analysis (pooled RR, 3.13; 95% CI, 0.68–14.30). The lack of statistical significance is likely attributable to the substantial heterogeneity among studies and the limited number of available investigations. Nevertheless, the overall body of evidence suggests that maternal metabolic disturbances during pregnancy may contribute to long-term neuroimmune susceptibility in offspring. Further large-scale, well-designed prospective cohort studies with detailed characterization of diabetes subtype, glycemic control, and relevant confounding factors are needed to clarify the nature of this association, elucidate the underlying biological mechanisms, and identify potential opportunities for early-life prevention of MS.
